# Prediction and Interaction in Complex Disease Genetics: Experience in Type 1 Diabetes

**DOI:** 10.1371/journal.pgen.1000540

**Published:** 2009-07-03

**Authors:** David G. Clayton

**Affiliations:** Juvenile Diabetes Research Foundation/Wellcome Trust Diabetes and Inflammation Laboratory, Department of Medical Genetics, Cambridge Institute for Medical Research, Cambridge University, Cambridge, United Kingdom; University of Oxford, United Kingdom

## Introduction

Much of the public media discussion of genetics of common diseases has centered on opportunities for targeted preventive actions. At the same time, in the specialist literature, there has been extensive discussion of “interaction,” both between genes and between genes and environment. These two topics concern the use and interpretation of statistical models for risk of diseases with several, perhaps many, etiological risk factors, and were both the subject of lively debate some 30 to 40 years ago when such models first came into widespread use in epidemiology. Here these debates are revisited and illustrated with results from an analysis of the genetics of type 1 diabetes (T1D). Details of this analysis are provided in section 1 of Text S1.

## Prediction

**Figure 1:**
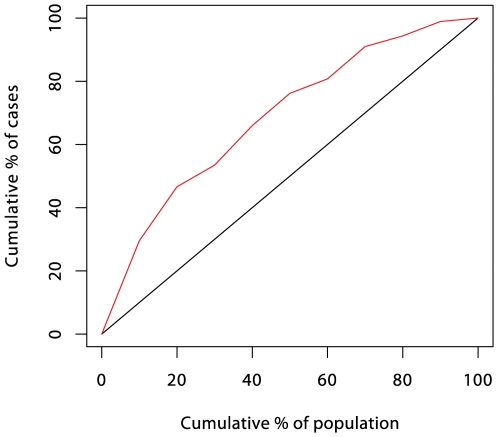
ROC curve for the prediction score of Truett, Cornfield, and Kannel [2] (five-year incidence of coronary heart disease in the Framingham cohort study).

Attempts to predict risk of disease from multiple risk factors began in the early 1960s, mainly in the context of coronary heart disease [1]. The logistic regression model soon became the method of choice, an early example being the five-year coronary disease risk score calculated from the Framingham cohort study data [2]. Predictive power of such models is often summarized by a receiver operating characteristic (ROC) curve; subjects are ranked in descending order of their predicted risk and the cumulative proportion of subjects who eventually succumb (cases) is plotted against the corresponding cumulative proportion of the population. Figure 1 illustrates such a plot using the Framingham data as an example; 29.6% of cases fell in the highest decile of predicted risk in the population, 46.6% fell in the top quintile, and so on. In terms of the use of the prediction score in a screening test, the ROC curve plots the sensitivity against (one minus) the specificity [3] for all possible thresholds for the score. A third measure of the accuracy of a screening test is the positive predictive value (PPV), the proportion of screen-detected patients who will go on to develop disease. For a rare event such as serious disease incidence, the ratio of true positives to false positives, PPV/(1−PPV), is given by multiplying the population risk by the ratio of the ordinate to the abcissa of the ROC curve. Although there have been many attempts to represent predictive efficacy in terms of a single number [4], such indices are often misleading, and it will generally be necessary to consider the whole curve when assessing the usefulness of prediction for clinical or public heath purposes.

Initial hopes that multivariate risk scores could form the basis of a prevention program based on targeted intervention in high-risk subjects quickly foundered owing to inadequate prediction. Rose [5] eloquently described the difficulty as the “prevention paradox,” in which “a large number of people at low risk may give rise to more cases of disease than the small number who are at high risk.” In terms of the ROC curve, the problem is that the ratio of ordinate to abcissa is usually only high enough to achieve an acceptable PPV at the very high end of the spectrum of risk, and this contributes a relatively small proportion of total cases. With the exception of screening for presence of early-stage disease, these arguments led to a swing away from the strategy of targeted intervention in favor of preventive strategies aimed at entire populations.

Interest in the possibility of individualized approaches to prevention and treatment has recently been reawakened in the context of advances in genetics. For example, Sir George Radda, then chief executive of the Medical Research Council, stated: “In 20 years' time, we may see individualized approaches to disease prevention and treatment” [6]. Ironically, such public pronouncements came at a time when complex disease genetics seemed to be making little headway [7]. Recent successes of genome-wide association studies have established a more optimistic climate of opinion, but it remains unclear whether such advances have the potential to deliver sufficiently accurate predictions to make targeted intervention a realistic possibility. In common with many such statements, Radda's remarks bracket prevention and treatment, but there are important differences, notably in the frequency of outcomes and in the need for high PPVs. While, in the treatment of disease, inaction is rarely an option and any prediction, however imperfect, may lead to benefit to patients, a preventive strategy based upon targeting high-risk subgroups will usually require more accurate prediction in order to be both ethical and effective from a public health standpoint.

**Figure 2:**
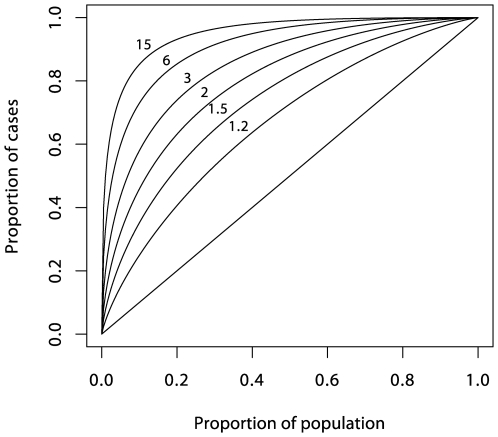
Theoretical ROC curves for various values of *λ_s_* under the polygenic multiplicative model.

It now seems that most genetic associations for common diseases currently being discovered are weak and, taken alone, would provide limited prediction [8]–[10]. However, a more open question is whether prediction would be adequate if all relevant genetic loci were eventually identified. This depends on the heritability of the condition and the model for risk. In the special case of many loci acting multiplicatively as in the logistic regression model, the ROC curve for prediction from a set of loci can be deduced from the sibling recurrence risk *λ_s_* [11] (see also section 2 of Text S1). Figure 2 shows a series of such curves. The most extreme curve corresponds to *λ_s_* = 15, typical of values quoted for autoimmune diseases such as T1D, multiple sclerosis, and Crohn disease. This assumes that all of the reported *λ_s_* is attributable to genetics rather than shared environment and that all relevant loci have been identified. Yet for diseases with cumulative incidence below 1%, even this would fail to deliver high PPV together with high sensitivity. For diseases such as type 2 diabetes and ischemic heart disease, for which reported values of *λ_s_* are three or less, much of which may be attributable to shared environment, the ROC curves suggest that individual prediction will be extremely poor, even if all loci could be identified and taking account of the rather greater frequency of such conditions in the population. The more extravagant claims for the utility of genetics in targeted prevention would therefore seem implausible, although it has been suggested that genetic information may have a more limited role in more effective delivery of screening programs [11].

### T1D Analysis

**Figure 3:**
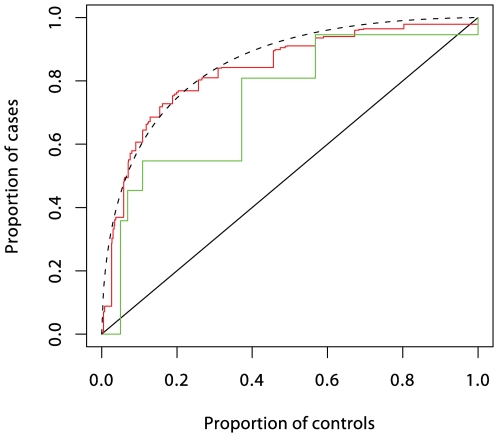
ROC curves prediction from loci in the MHC region. Prediction using six single nucleotide polymorphisms (SNPs) is shown in red, while prediction using HLA-DRB1 is shown in green. These curves (and those in Figures 4 and 5) were obtained by fitting logistic regression models as described in Text S1 and calculating the proportions of cases and controls with prediction scores exceeding each possible value. The dashed curve corresponds to the theoretical curve for the polygenic multiplicative model with *λ_s_* = 3.13.

**Figure 4:**
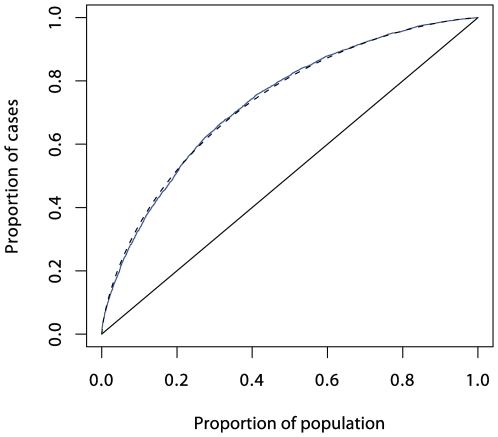
ROC curve prediction from the SNPs outside the MHC region listed in Supplementary Table 1 in Text S1 (in blue). The dashed curve corresponds to a polygenic multiplicative model with *λ_s_* = 1.48.

**Figure 5:**
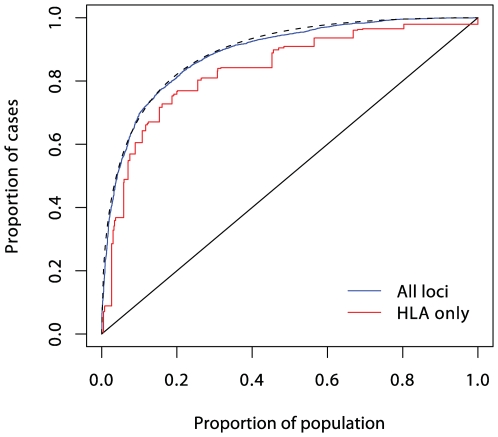
ROC curve prediction from all the SNPs listed in Supplementary Table 1 in Text S1 (in blue). The prediction curve using the six MHC SNPs alone is shown in red, and the dashed curve corresponds to a polygenic multiplicative model with *λ_s_* = 4.75.

Understanding of the genetic determinants of T1D commenced with the discovery, in 1973, of a strong HLA association [12]. This was followed, in 1984, by discovery of the INS gene association [13]. Subsequent progress was slow, resulting in the discovery, by 2007, of only three further associated loci in candidate genes. However, the advent of genome-wide association studies has resulted in an explosion of new discoveries, with more than 40 disease susceptibility loci now identified [14]. The impact of these discoveries on prediction are displayed in Figures 3, 4, and 5. Although the new discoveries will have undoubted value for our understanding of the disease, their impact on prediction is modest.

Current known loci explain a *λ_s_* of just under five, as compared with the value of 15 often quoted. However, it is likely that the latter figure is exaggerated, and the *λ_s_* attributable to inheritance is likely to be less than ten. The heritability explained will be increased to some degree when the known regions are more fully studied, but the bulk of the remaining heritability is likely to be attributable to many small (or rare) effects, most of which are unlikely to be mapped. Thus, even for this highly heritable disease, the prediction achievable could fall some way short of that required for a targeted prevention strategy.

## Interaction

This topic has received much recent attention, but with scant reference to the lively debate of the early 1980s, which was initiated in response to widespread overinterpretation of “interaction” in logistic regression models. It has been widely noted that statisticians and biologists attach different meanings to the word “interaction” [15]–[19]. Whereas a biologist would use the word (often loosely) to describe an aspect of biological mechanism, for a statistician, interaction between two factors represents deviation from some mathematical model for joint effects of several factors on risk. It only has an interpretation for mechanism if the mathematical model tested has an interpretation. This is rarely the case; most mathematical models are convenient fictions and would certainly be rejected given sufficient sample size. Writing in 1991, Thompson [20] noted that, although much of the debate had by then subsided, few clear conclusions had emerged. His review concluded: “[C]hoice among theories of pathogenesis is enhanced hardly at all by the epidemiological assessment of interaction…What few causal systems can be rejected on the basis of observed results would provide decidedly limited etiological insight.” In following years, this has been the consensus view among epidemiologists—a fact that renders the recent re-emergence of interest in genetic epidemiology somewhat surprising.

In genetics, the confusion between statistical and biological notions of interaction goes back to Fisher's 1918 paper [21] in which he used the term “epistacy” to describe statistical interaction between different loci—a use to which a referee, R. C. Punnett, objected [22]. The confusion of which Punnett warned was further increased as Fisher's term “epistacy” became widely replaced by Bateson's term [23], “epistasis,” which inspired it [24]–[27]. The difference between Fisher's and Bateson's use of these terms illustrates a distinction that statisticians draw between “quantitative” interaction and “qualitative” interaction. In a quantitative interaction, presence of one factor is associated with a larger or smaller effect of a second factor, but the direction of effect is unchanged. The presence of interaction then depends on the way “effects” are measured; if there is no interaction when effects are measured by relative risks (as in the logistic regression model), there would be interaction if effects were to be measured by differences in risk (as in an additive risks model). And vice-versa. This ambiguity contrasts with qualitative interaction, where one factor reverses the direction of effect of the other or, as in Bateson's epistasis, when presence of one factor simply negates the effect of another.

While qualitative interaction has clear implications for mechanism, conventional statistical tests for interaction do not test for this. A test for reversal of direction of effect has been proposed [28], but is rarely used, and it is anyway arguable whether such effects will be widespread in the epidemiology of common diseases. Masking of effect is perhaps more plausible, but it could be argued that formal proof of this is impossible since this would require proof of the hypothesis of no effect in a subgroup. However, Berrington de González and Cox [29] argued that to take the position that only effect reversal provides evidence of biological interaction risks overlooking important findings. In practice, the size of effects is crucial. In experiments with congenic strains of mice, observed effects are often so large that it can be reasonable to infer their absence when they are not observed. In the context of T1D, such work has recently been reviewed by Ridgway et al. [30], who concluded: “Using congenic mice, gene–gene interactions and gene masking effects have been observed that make large impacts on the T1D frequency whereas these effects are mostly hidden in a genetically segregating population such as a backcross one or an F2 generation, or in conventional genetic association studies in humans.”

A model, it has been claimed, that does allow a biological interpretation of quantitative interaction is the additive model for risk, which corresponds (to a close approximation) with the model of independent sufficient causes [31] (for an alternative view of this model see [32]). Often, however, the model of independent causes will be implausible a priori, and its rejection would provide a “decidedly limited etiological insight.” It often provides a decidedly poor fit to empirical data, and the model usually preferred is the logistic regression model, in which the odds in favor of developing disease (proportional to the risk, for rare diseases) is given by the product of multiplicative effects or, equivalently, by additive effects on the log odds scale. To avoid confusion, the term “additive model” will henceforth refer to the model in which effects are additive in risk. Unlike the additive risks model, the logistic model has no simple biological interpretation and is useful only in so far as it provides an empirical description of real phenomena, and quantitative interaction in this model will rarely have a biological interpretation.

Despite the problems of interpreting tests for quantitative interaction, statistically significant results are often heralded as “significant” in a wider sense. An example is the much cited work concerning interaction of life stress and a polymorphism of the *5-HTT* gene on depression [33]; although only quantitative interaction tests are quoted, the figures shown are suggestive of qualitative interaction (even though these only show fitted values from regression equations). It seems unlikely that the model of independent causes would have fitted these data, but one might question whether the a priori support for this model would render its falsification anything other than a “decidely limited etiological insight.” Additional evidence for the widespread overinterpretation of quantitative interaction can be found in the literature describing calculation of sample sizes necessary for its detection [34]–[36].

Another reason for recent interest in gene–gene interaction concerns its implications for association studies. It is argued that the genetic effects currently being detected are small, but that interaction between genes is likely to be ubiquitous. From these tenets it is concluded that larger effects (and better prediction) will be seen if we study genes two or more at a time. A similar argument has been influential in generating interest in gene–environment interactions. The effects of environmental and behavioral factors on disease risk are typically stronger than the effects of genetic loci, but measuring them in free-living populations is difficult and prone to the well-documented problems of bias, confounding, and reverse causality. It has been argued that research into such influences has reached its limits, effect sizes being small in comparison with methodological errors [37]. Again a powerful intuition is that, since genes and environment must interact, larger effects will be found in genetically at-risk subgroups of the population. However, such arguments confuse statistical and biological interaction; the fact that gene–gene and gene–environment interaction, in the mechanistic sense, are probably widespread does not mean that statistical interaction in the logistic regression model will be equally widespread.

The possible role of interaction in the detection of new associations is stressed in emerging writings of computer scientists. These approaches use measures of “synergy” derived from information theory [38],[39]. Synergy/interaction is judged to be present when higher dimensional contingency tables carry more information than their lower order margins. The precise measure of information synergy proposed by these authors can be criticized, but is quite close to a measure of deviation from multiplicative effects. It can be argued that a more satisfactory treatment [40] leads to a definition of information theoretic synergy that is precisely the same as interaction in the logistic regression model (see section 3 of Text S1). Thus, entropy measures of synergy differ little from standard tests for statistical interaction in the logistic model and suffer the same problems of interpretation. But advocates of this approach have not been immune to the tendency to confuse mathematical and biological notions of interaction. For example, Moore et al. wrote [38]: “It is the promise of systems biology to deliver an etiological understanding of epistasis.” There is often a strong implication that genes that act synergistically in this information theoretic sense act in the same causal pathway—an assumption that cannot be justified rigorously.

In complex disease genetics, models for additive and multiplicative contributions to risk have both been discussed in some detail. In the context of affected relative pair linkage studies, Risch [41] considered the additive model for risks as a close approximation to the idea of “genetic heterogeneity.” In contrast, he proposed the multiplicative model for risks as a model for epistasis and demonstrated that, under this model, recurrence risks fall away much more rapidly with increasing distance of relationship than under the additive model—as is observed for most common complex diseases. Confusingly, in the literature on association studies, epistasis is more commonly identified with deviation from the multiplicative model. Epistasis has also been defined in terms of departure from the multiplicative model for fitness in population genetics [27], motivated by the same mathematics that underlies the case-only test for gene–gene interaction [42]: i.e., that under this model, loci that are statistically independent in the population remain so in cases.

Estimation of the joint effects of multiple genes, or of genes and environment, remains an important aim, but interpretation of statistical tests for presence or absence of interaction are problematic. The T1D example discussed below demonstrates this.

### T1D Analysis

The interaction between HLA and *PTPN22* illustrates the problem of interpretation. As in previous reports [14], [43]–[46], the effect of *PTPN22*, measured by relative risks, is greatest in the low-risk HLA group (shown as the first entry in each section of Table 1). This variation in relative risks defines interaction in the context of the multiplicative model, and is measured by the “interaction” parameters—the ratios of relative risks shown as the second entry in each section of the table. However, when main effects are included and the results converted to absolute risks (the final two entries in each section of the table), it can be seen that the additional risk due to *PTPN22* is largest in the high-risk HLA group. Since the risk differences are not constant, there would also be said to be interaction in the context of the additive model, but it is in the reverse direction. Neither the additive nor the multiplicative model describe the joint action of these two loci. Whereas deviation from the additive model can be interpreted as rejection of the model of independent sufficient causes, rejection of the multiplicative model has no biological interpretation of which the author is aware.

**Table 1:** T1D risk by HLA and rs2476601 (*PTPN22*) genotype.

HLA Risk	rs2476601 Genotype		
	GG	AG	AA
Low	*1.0*	2.56	6.76
	*1.0*	*1.0*	*1.0*
	*1.0*	2.56	6.76
	1.7	4.4	11.6
Medium	*1.0*	2.29	3.24
	*1.0*	0.89	0.48
	4.02	9.20	13.04
	6.9	15.8	22.4
High	*1.0*	1.67	2.37
	*1.0*	0.65	0.35
	31.24	52.07	74.03
	53.6	89.3	127.0

HLA risk is grouped by tertiles of a prediction score using the six MHC SNPs listed in Supplementary Table 1 in Text S1. The first entry in each section shows effects of rs2476601 for each HLA group, expressed as relative risks with the “GG” genotype as reference (shown in italic). The second line in each section shows these expressed relative to the low- risk HLA group. The four ratios of relative risks that remain after sweeping out the first row and column are the interaction parameters in the logistic regression model, and the test for interaction is a test of the hypothesis that these do not differ from unity. These “ratios of ratios” are difficult to interpret, but are the only parameters identifiable from case-only data. The third entry in each section shows joint effects of HLA and *PTPN22* when main effects are included, and the final line expresses these as absolute risks per 1,000 in a population in which the overall cumulative incidence is 1%.

In logistic regression analysis of the T1D data, there are many interactions that achieve nominal (*p*<0.05) levels of significance. But, with the exception of strong interactions within the major histocompatibility complex (MHC), these interactions are small and have a modest effect on prediction, and their omission leads to scarcely perceptible loss of prediction. For example, the area under the ROC curve for prediction using non-HLA loci and allowing for interactions (Figure 4) is 0.738, and this falls only to 0.733 when all interaction terms are omitted.

Further analysis shows that the model for additive accumulation of genetic risks for T1D can be rejected beyond doubt, but the multiplicative model, while not perfect, provides a remarkably good approximation.

## Conclusion

Many authors have recently commented on the modest predictive power of the common disease susceptability loci currently emerging. However, here it is suggested that, for most diseases, this would remain the case even if all relevant loci (including rare variants) were ultimately discovered. It must also be said that similar difficulties are faced when making predictions on the basis of environmental risk factors, as was recognized by epidemiologists more than 30 years ago. Prediction at the individual level is an ambitious aim, particularly in the context of disease prevention.

Similarly, the recent interest in interaction in genetics has also been characterized by exaggerated expectations for the inferences that can be drawn from epidemiological data. These, too, were widely prevalent in epidemiology thirty or more years ago, but have since given way to more limited expectations; aside from rejection of a model in which two factors operate through wholly unrelated mechanisms, little can be deduced about mechanism from the observation of statistical interaction—particularly when effects are not large.

## Supporting Information

Text S1 Prediction and interaction in complex disease genetics: experience in type 1 diabetes.
(0.15 MB PDF)
