# Supplementary material for: Prediction and Interaction in Complex Disease Genetics: Experience in Type 1 Diabetes
Source: PLoS Genet. 2009 Jul 3;5(7):e1000540. doi: 10.1371/journal.pgen.1000540 (PMC2703795; doi:10.1371/journal.pgen.1000540)
Supplement: Text S1 — Prediction and interaction in complex disease genetics: experience in type 1 diabetes. (0.15 MB PDF) [file pgen.1000540.s001.pdf]

Prediction and interaction in complex disease genetics: experience in type 1 diabetes. Supplementary information

1 T1D analysis

This section describes the analysis of the T1D data in more detail. Unless otherwise stated, table and figure numbers refer to the main paper.

A list of the currently most associated SNPs in the 40+ regions now know to be associated with T1D is shown in Supplementary Table 1, although most of the SNPs which lie outside the MHC region have a rather small effect on the risk of T1D (Supplementary Figure 1). This section presents details of the analysis relating T1D risk to all of these known loci. This analysis has been carried out in up to 9,000 cases and 11,000 controls drawn from throughout Great Britain. This collection is described elsewhere [1]. Some of these data formed part of the datasets which initially implicated some of the loci, so that there may be a small exageration of predictive power due to the "winner's curse". However, the inclusion in the model of only those loci which achieved very stringent levels of statistical significance and were replicated in further samples is a somewhat conservative strategy.

HLA effects

The relationship between HLA loci and the risk of T1D is complex and still somewhat controversial, with associations reported with *HLA-DRB1*, *HLA-DQB1*, *HLA-A* and *HLA-B* [2]. The MHC region is extremely polymorphic and exhibits strong linkage disequilibrium and, as a result, the haplotype analyses which have dominated the field are complicated by problems of multiplicity. For these reasons, and because the HLA associations are not a major focus of this review, for the main analysis the HLA effect will be, as far as possible, captured using the six SNPs chosen in the recent genome-wide association study [3] using the Illumina 550K array. These SNPs are listed in the lower section of Supplementary Table 1, and all six were successfully typed in 3,997 of our cases and 3,972 of our controls. A logistic regression analysis was carried out, including terms in the order

1. "allelic" effects, entering each SNP as a numeric variable coded 0,1 or 2,
2. "dominance" effects, entering binary variables coding SNPs as homozygous or heterozygous, and
3. terms coding statistical interaction between loci.

Inclusion of dominance and interaction terms was decided on the basis of improvement in Akaike's information criterion (AIC) [4]. There were large dominance and interaction effects in this analysis as would be expected given the pattern of association shown, for example, for *HLA-DRB1* (Supplementary Table 2).

Figure 3 shows ROC curves for the predictions using six SNPs and using *HLA-DRB1* alone. The λ_S attributable to *HLA-DRB1*, based on the relative risks shown in Supplementary Table 2, is 2.31.

SNP	Band	Proximal gene(s)
rs2476601	1p13	*PTPN22*
rs2816316	1q31	
rs3024505	1q32	*IL10*
rs1534422	2p25	
rs917997	2q12	*IL18RAP*
rs1990760	2q24	*IFIH1*
rs7574865	2q32	*STAT4*
rs3087243	2q33	*CTLA4*
rs333	3p21	*CCR5*
rs10517086	4p15	
rs2069763	4q27	*IL2*
rs2069762	4q27	*IL2*
rs6897932	5p13	*IL7R*
rs11755527	6q15	*BACH2*
rs9388489	6q22	*TNFAIP3*
rs10499194	6q23	*TNFAIP3*
rs6920220	6q23	
rs1738074	6q25	*TAGAP*
rs7804356	7p15	*SKAP2*
rs4948088	7p12	
rs7020673	9p24	*GLIS3*
rs12722495	10p15	*IL2RA*
rs2104286	10p15	*IL2RA*
rs11594656	10p15	*IL2RA*
rs947474	10p15	*DKFZp667F0711*
rs10509540	10q23	

SNP	Band	Proximal gene(s)
rs689	11p15	*INS*
rs4763879	12p13	*CD69*
rs2292239	12q13	*ERBB3*
rs3184504	12q24	*SH2B3*
rs1465788	14q24	*C14orf181*
rs4900384	14q32	
rs3825932	15q25	*CTSH*
rs12708716	16p13	*CLEC16A*
rs12444268	16p12	*UMOD*
rs4788084	16p11	
rs7202877	16q23	
rs2290400	17q12	*GSDMB*
rs45450798	18p11	*PTPN2*
rs478582	18p11	*PTPN2*
rs763361	18q22	*CD226*
rs425105	19q13	*PRKD2*
rs2281808	20p13	*SIRPG*
rs3788013	21q22	*UBASH3A*
rs5753037	22q12	
rs229541	22q13	*C1QTNF6*
rs2664170	Xq28	*GAB3*
rs805294		(MHC)
rs2187668		(MHC)
rs9275313		(MHC)
rs9275388		(MHC)
rs9275425		(MHC)
rs9275614		(MHC)

Supplementary Table 1. SNPs currently most strongly associated with T1D (see `http://www.t1dbase.org`). The final group of 6 SNPs were chosen to capture the HLA associations.

Calculation of λ_S attributable to the six SNPs is problematic because of the strong dominance and interaction effects. However a polygenic multiplicative model with $\lambda_S = 3.1$ fits the observed ROC closely and it is reasonable to assume that this approximates the λ_S explained by this association. This agrees closely with an estimate of the λ_S attributable to HLA from estimates of IBD sharing derived from linkage studies. Using data then available Risch, in 1987 [5], estimated this to be 3.42. More recent and more extensive data, for 1,967 affected sib–pairs with both parents typed [6] yields an estimate of $\lambda_S = 3.07$. It would seem, therefore, that rather few SNPs can capture most of the heritability of T1D risk attributable to HLA associations.

Loci outside the MHC region

At least 40 of the 48 non-MHC SNPs listed in Supplementary Table 1 have been typed for 7,198 of the available cases and 7,764 of the controls. In these subjects, the small number of failed genotypes were imputed and, using the same procedure as described above, a logistic regression model was used to predict disease status. The resulting ROC curve is shown in Figure 4. Also shown is the best fit ROC for a polygenic multiplicative model, which has $\lambda_S = 1.48$.

Despite the excellent fit of the multiplicative model, the final fitted model involved a number of

Genotype	Cases	Controls	Odds ratio
X/X	193	1,606	(Reference)
$3/X$	488	728	5.6
$4/X$	933	978	7.9
3/3	343	72	39.6
4/4	332	147	18.8
3/4	1,276	185	57.4
Total	3,565	3,716	

Supplementary Table 2. T1D risk and *HLA–DRB1* genotype. X represents alleles other than 3 or 4.

dominance and interaction terms. For 15 SNPs, the model of multiplicative allelic effects was rejected in the final model, which also included 174 first order interaction terms. However, the Akaike criterion for inclusion of extra terms is a lax one (even less demanding than a 5% significance level) and the sample size is extremely large, and all these additional terms were extremely small. The ROC curve for the model in which all loci act multiplicatively and each locus has multiplicative allelic effects is indistinguishable from that shown in Figure 4, giving only minimally reduced prediction (equivalent to $\lambda_S = 1.46$).

Overall prediction

Finally, the regression analysis was repeated using all the 54 SNPs listed in Supplementary Table 1. The resulting ROC is shown in Figure 5, together with that for the best-fitting ROC for a multiplicative polygenic model, which has $\lambda_S = 4.75$. This agrees closely with the product of values attributable to HLA ($\lambda_S = 3.12$) and to other loci ($\lambda_S = 1.48$) — consistent with approximately multiplicative effects although, again, the final model included many terms representing deviations from a purely multiplicative model, the larger terms tending to be interactions with HLA loci. Such interactions have been reported previously, notably an interaction between HLA and *PTPN22* [3, 7–10].

Interaction

The analyses presented above all use the logistic regression model, which closely approximates the model of multiplicative effects of loci upon risk. Many interactions achieved nominal statistically significance ($P <$ 0.05 or better) but were small and had an almost imperceptible impact the ROC curves. The difficulty in drawing any clear biological interpretation from these interactions is illustrated by the previously reported interaction between *PTPN22* (here represented by the SNP rs2476601) and HLA (here measured by a risk score calculated from six SNPs). In these data this interaction was significant in a case–only test ($p = 0.004$) and negative, indicating that the effect of the *PTPN22* SNP is smallest when the HLA risk is highest. Since this test is a test for departure from the multiplicative model, "effect" in this context is measured by the relative risk. This is illustrated in the fisrt entries in the cells of Table 1 and the parameters tested in the formal interaction test are shown as the second entries. A different perspective is gained when, in the third and fourth entries, we examine the joint effect of both loci and their predictions for absolute risk. From these tables it is evident that, although the *relative* effect of *PTPN22* is greatest in the low HLA risk group, its *absolute* contribution to risk is greatest in the high HLA risk group.

The existence of interaction terms does, however, beg the question whether a better model could be fitted. Although it is clear from the above example that an additive model for risk is unlikely to fit these data, this is a possibility that might wish to be considered in other contexts. The standard method for choosing between additive and multiplicative models is to embed both models in a wider class. Thus, instead of the logistic regression model, consider the more general model:

$$g\left(\Pr(\text{Disease}); \rho\right) = \beta_0 + \beta_1 x_1 + \beta_2 x_2 \cdots$$

where the parameter ρ controls the scale on which risk contributions accumulate. A convenient choice for the "link" function, $g()$, is powers of the odds:

$$\begin{aligned} g(p;\rho) &= \frac{1}{\rho}\left\{\left(\frac{p}{1-p)}\right)^{\rho} - 1\right\} && (\rho \neq 0), \\ &= \log(\frac{p}{1-p}) && (\rho = 0). \end{aligned}$$

This model reduces to the multiplicative model when $\rho = 0$ and to the additive model when $\rho = +1$. Negative values of ρ represent models in which risks accumulate faster than multiplicatively. This model, without explicit interaction terms, is fitted for a range of values of ρ and the fit assessed by examining the (log) likelihood profile. These curves are shown in Supplementary Figure 2 using non-HLA SNPs (blue curve) and, with a smaller sample size, using all SNPs (red curve). Although a common experience in epidemiology has been that it can be difficult to discriminate between additive and multiplicative models for risk, the combination of strong prediction and large sample sizes mean that there is no such difficulty here. The additive model for risks, $\rho = 1$, is such a poor fit that it could not be fitted with the available software, and the range of supported models differ only slightly from the multiplicative model, $\rho = 0$ (conventional 95% confidence limits for ρ correspond approximately with values of ρ for which the log likelihood ratio is greater than -2). In the case of the model for all SNPs, this excludes the multiplicative model, the best choice being consistent with the joint effect of two loci being being more than the product of their single effects. However this is entirely due to dominance and interaction within the MHC region. When these terms are included in the model, the pattern that emerges (green curve) is that there is a highly significant, though small, shift towards a model in which joint effects are slightly less than the product of single effects. This would suggest that smaller relative risks will tend to be observed for new loci in high risk populations than in low risk populations, and this has been advanced as an explanation for the observation of rather lower effect sizes in multi-case families than in sporadic cases [3, 11]. However, it should be stressed that the shift away from the multiplicative model is very small and the predictions of the best fit model are virtually indistinguishable from that of the logistic regression model.

Heritability

The analysis presented above suggests that known T1D susceptibility loci account for a sibling relative recurrence risk, λ_S, of just under 5. This compares with the figure of 15 widely quoted in the literature (see, for example, Risch [5]). There are at least three possible explanations for this discrepancy:

1. a large fraction of the genetic influences on T1D have yet to be discovered, or
2. reported values of λ_S are biased, or
3. observed values of λ_S are partially due to shared environmental influences.

If λ_S attributable to genetic influences really is 15, then yet undiscovered loci would, together, need to have an effect at least as strong as HLA and three times as strong as that of all the remaining known loci. There must be many undiscovered loci associated with T1D. The majority of these will have even smaller effects than those already discovered. A few may have larger effects, but have not been discovered because they are not tagged by the current generation of genome-wide SNP chips. This latter group includes common copy number variants, not all of which will be tagged, and low-frequency variants. There is considerable interest in copy number variation at present, and it will not be too long before we know whether common copy number variants play a role in T1D. Likewise, advances in high-throughput sequencing will allow us to search for low frequency variants, albeit not yet on a genome-wide scale. However the now extensive linkage evidence would suggest that new disease susceptibility loci are not sufficiently strong, or sufficiently concentrated in specific regions, to yield large local contributions to λ_S.

A sobering example is that a variant with frequency of 1% conferring a relative risk of 2 only generates a λ_S of 1.01 so that around ten such loci in a gene would be required to generate a λ_S of 1.1. Undoubtedly, lower frequency disease susceptibility variants will be present in regions already discovered, and these will enhance their effects. However, in a recent study of ten candidate genes, Nejentsev *et al.* [12] found low frequency variants with large effects in only one gene. To summarise, if $\lambda_S = 15$ is an accurate estimate of the heritability of T1D much, if not most, of the remaining variation will be distributed as small effects or rare variants throughout the genome. The cataloguing of all this variation would be a daunting task, even if suitable methodology were available.

It could be, however, that λ_S has been exaggerated. Perhaps the most comprehensive study of recurrence risk in siblings of T1D cases has been carried out in Finland [13]. This suggested that cumulative incidence by age 50 in siblings of a case of T1D was 6.9%, of which just over 3% was by age 15. Comparable population data is not readily available, but a review in 1993 [14] quoted the incidence rate at ages 0-15 in Finland as 35.3 per 100,000 person-years, yielding a cumulative incidence by age 15 of 0.53%. These figures would suggest a value for λ_S closer to 6 than to 15. However, there are strong secular trends in the incidence rate and a more careful analysis would be necessary to obtain an accurate estimate.

An alternative measure of the heritability of T1D is the ratio of incidences between monozygotic (MZ) and dizygotic (DZ) twins. Arguably, this measure is less contaminated by the effects of shared environment, although an effect of shared placenta in MZ twins is not beyond the bounds of possibility. A recent study in Finland [15] estimated the probandwise concordance in MZ twins, which can be taken as an estimate of λ_{MZ}, at 42.9% while the same index for DZ twins was 7.4%. This yields a ratio of 5.8. An earlier study of Danish twins [16] estimated crude probandwise concordance rates as 53% in MZ twins and 11% in DZ twins. An analysis which estimated cumulative incidence by age 35 yielded, respectively 70% and 13%. Thus the Danish data are consistent with a $\lambda_{MZ} : \lambda_{DZ}$ ratio of around 5. Numbers are small in these studies, but together they are consistent with the $\lambda_{MZ} : \lambda_{DZ}$ ratio being in the region 5 to 6. Under the polygenic multiplicative model, the ratio of recurrence risks of MZ to DZ twins would be the same as λ_S (see below), although it will be rather less under models with strong dominance effects. Thus, the linkage analysis referred to above estimates the $\lambda_{MZ} : \lambda_{DZ}$ ratio attributable to HLA to be 2.10 (compared with 3.07 for λ_S) which, assuming that non-HLA and HLA effects combine approximately multiplicatively, would suggest that non-HLA loci account for a $\lambda_{MZ} : \lambda_{DZ}$ ratio of between $5/2.1 = 2.4$ and $6/2.1 = 2.9$. Assuming that the polygeneic multiplicative model is a reasonably accurate model for the effects of all the non-HLA loci, the λ_S attributable to these will be approximately the same as their contribution to $\lambda_{MZ} : \lambda_{DZ}$. Thus, we might expect the overall λ_S to lie between $3.07 \times 2.4 = 7.4$ and $3.07 \times 2.4 = 8.9$ — a slightly larger estimate than given by the sibling studies, but still substantially smaller than the value of 15 often quoted. However, all estimate remain appreciably above the value of 4.75 currently explained. It is entirely plausible that disease susceptibility variants which are either too rare of have too small an effect size to be detected by current epidemiological methods explain the residual heritability.

2 The ROC curve and λ_S in the polygenic multiplicative model

This derivation closely parallels that of Pharaoh *et al.* [17], although here the relationship with logistic regression models is rather more explicit.

Let $x_\ell; \ell = 1 \ldots L$ denote the number of copies (0, 1 or 2) of loci carried by an individual at L loci. The fully multiplicative model in which each copy of an allele at locus ℓ multiplies risk by $\exp \beta_\ell$ and effects of different loci combine multiplicatively has

$$\begin{aligned} \Pr(\text{Disease}|\text{Genotype}) &= e^{\eta}, \\ \eta &= \beta_0 + \sum_{\ell=1}^{L} \beta_\ell x_\ell. \end{aligned}$$

When Pr(Disease|Genotype) is small, this is approximately the same as the logistic regression model, which has the advantage that the parameters measuring effects of genotype are constant (in expectation) under case–control sampling. If L is large, the distribution of the risk scores, η, in the population will be approximately normal, let us say with mean μ and standard deviation σ. Then it is easily shown that the distribution of the risk score in cases of disease is also normal with standard deviation σ, but with mean $(\mu + \sigma^2)$. Given σ, this defines the ROC since this does not depend on μ.

The population risk is given by the expectation of e^η in the population, which may be shown to be

$$K = \mathbb{E}(e^\eta) = \exp(\mu + \sigma^2/2).$$

Now consider two potentially related individuals in this population, and denote their risk scores by η_1 and η_2. (η_1, η_2) is drawn from a bivariate normal population in which both marginal means are μ, both marginal standard deviation are σ, and the correlation coefficient is ρ. The probability that they are both cases is given by the expectation of $e^{\eta_1+\eta_2}$, which can be shown to be

$$\mathbb{E}\left(e^{\eta_1+\eta_2}\right) = \exp(2\mu + \sigma^2 + \rho\sigma^2).$$

The relative recurrence risk for relatives of type R is then given by division of this expression by K^2:

$$\lambda_R = \frac{1}{K^2}\exp(2\mu + \sigma^2 + \rho_R\sigma^2) = \exp(\rho_R\sigma^2)$$

where ρ_R is the correlation between risk scores, η, for relatives of type R. In outbred populations this correlation is simply twice the kinship coefficient. Thus the logarithm of the relative recurrence risk is directly proportional to the kinship coefficient[1]. The implied ROC for given λ_S can be calculated by noting that $\rho_S = 0.5$, so that

$$\sigma^2 = 2\log\lambda_S.$$

For twin recurrence risks, these results yield:

$$\begin{aligned}\lambda_{MZ} &= \exp(\sigma^2),\\ \lambda_{DZ} &= \exp\left(\frac{1}{2}\sigma^2\right)\end{aligned}$$

so that

$$\frac{\lambda_{MZ}}{\lambda_{DZ}} = \exp\left(\frac{1}{2}\sigma^2\right) = \lambda_S.$$

3 Entropy and synergy

In information theory, entropy is a measure of uncertainty associated with a probability distribution. If a random variable, D, takes on possible values $d_i, \ldots, d_n$, then the entropy measure of uncertainty concerning D is

$$H(D) = \sum_{i=1}^{n} P(D = d_i)\log P(D = d_i).$$

In the context of this paper, D is disease status, taking on just two values — present or absent. If D is related to a second variable, for example a genotype, G, then knowing the value of this variable (g say) will reduce the remaining uncertainty to

$$\sum_{i=1}^{n} P(D = d_i|G = g)\log P(D = d_i|G = g).$$

[1]From this result it also follows that, when two loci act multiplicatively (so that there effects are additive on th elog risk scale), they also contribute multiplicatively to recurrence risks

The *conditional entropy* is the expectation, or average, of this over all possible values of G:

$$H(D|G) = \sum_g P(G=g) \sum_{i=1}^{n} P(D=d_i|G=g) \log P(D=d_i|G=g).$$

The amount by which the entropy for D is reduced by knowledge of G provides a measure of the information gain:

$$\mathrm{IG} = H(D) - H(D|G).$$

In commonly used definitions of "synergy" of genes based on entropy (for example [18, 19]), synergy between two genes, G_1 and G_2, is defined in terms of the difference between the information gain from both genes and the sum of the information gains from each gene individually:

$$\begin{aligned}\{H(D) - H(D|G_1, G_2)\} - \{H(D) - H(D|G_1)\} - \{H(D) - H(D|G_2)\} = \\ -H(D|G_1, G_2) + H(D|G_1) + H(D|G_2) - H(D)\end{aligned}$$

The idea generalises to provide definitions of higher order synergy. This measure is superficially appealing and can be computed very rapidly, but has several difficulties. Firstly, it is not necessarily positive so that the total information contributed by G_1 and G_2 could be less than the sum of their individual contributions; it is an odd form of synergy in which to whole is *less* than the sum of its parts! A second problem is that the definition is not invariant under case–control sampling. Both of these difficulties are illustrated by the case where G_1 and G_2 are in linkage equilibrium and act multiplicatively on the risk of disease. Then, in a cohort study, G_1 and G_2 are marginally independent and, for a rare disease, they are also approximately independent conditional upon D. Thus, because the measure of synergy can also be expressed as

$$\{-H(G_1, G_2|D) + H(G_1|D) + H(G_2|D)\} - \{-H(G_1, G_2) + H(G_1) + H(G_2)\},$$

the difference between entropy measures of conditional and marginal association between G_1 and G_2, this is approximately zero. In a case–control study, however, G_1 and G_2 remain conditionally independent but are no longer marginally independent, and the above measure of synergy becomes negative.

Although proponents of this approach stress that it is model free, the most important difficulty with it is that it cannot fail to beg the question: what is "no synergy"? If we are to assert that two genes have a synergistic action we must define what we mean by saying that they are *not* synergistic; we must have a null hypothesis. The definition of synergy above, when set to zero, does not lead to any simply interpretable pattern of risks except in very special cases. While maintaining an information-theoretic approach, these difficulties can be resolved by tackling the problem from the standpoint of the null hypothesis. How much uncertainty about D would remain if we knew the two-way relationships between D and G_1 and D and G_2 but did not know the complete relationship between all three variables? If this could be defined, then the amount by which entropy is reduced by knowing the *joint* effect of the two genes provides a more satisfactory definition of synergy. Good [20] argued that the former quantity is the maximum value that the entropy can take over all possible three way distributions, $P(D, G_1, G_2)$, given the known two-way marginal distributions $P(D, G_1)$, $P(D, G_2)$, and $P(G_1, G_2)$. The new measure of synergy of information would then become:

$$-H(D|G_1, G_2) + \mathrm{Max}\,\{H(D|G_1, G_2)\,|\,P(D, G_1), P(D, G_2), P(G_1, G_2)\}$$

Good proposed the principle of maximum entropy as a general principle for generating null hypotheses, but discussed the case of log–linear models for contingency tables in some detail. The logistic regression model for a binary outcome (here disease status, D) and discrete predictor variables (genes, G_1, G_2) is a special case of such models and Good's results show that the null hypothesis leading to maximum entropy is precisely the model of no interaction between G_1 and G_2 in the logistic regression model for D. Thus, this arguably more satisfactory information theoretic approach effectively equates synergy of information with interaction in the logistic model.

References

1. Todd J, Walker N, Cooper J, et al. (2007) Robust associations of four chromosome regions from genome-wide analyses of type 1 diabetes. Nature Genetics 39: 857–864.

2. Nejentsev S, Howson J, Walker N, et al. (2007) Localisation of type 1 diabetes susceptibility to the major histocompatibility complex class I gene, HLA-A and HLA-B. Nature 450: 887–892.

3. Barrett J, Clayton D, Concannon P, Akolkar B, Cooper J, et al. (2009) A genome–wide association study and meta–analysis indicate that over 40 loci affect risk of type 1 diabetes. Nature Genetics In press.

4. Akaike H (1974) A new look at the statistical model identification. IEEE Transactions on Automatic Control 19: 716–723.

5. Risch N (1987) Assessing the role of HLA–linked and unlinked determinants of disease. American Journal of Human Genetics 40: 1–14.

6. Concannon P, Chen WM, Julier C, Morahan G, Edrich H, et al. (2009) Genome-wide scan for linkage in type 1 diabetes in 2,496 multiplex families from the Type 1 Diabetics Genetics Consortium. Diabetes In press.

7. Hermann R, Lipponen K, Kiviniemi M, Kakko T, Veijola R, et al. (2006) Lymphoid tyrosine phosphatase (LYP/PTPN22) Arg620Trp variant regulates insulin autoimmunity and progression to type 1 diabetes. Diabetologia 49: 1198–1208.

8. Steck AK, Liu SY, McFann K, Barriga KJ, Babu SR, et al. (2006) Association of the PTPN22/LYP gene with type 1 diabetes. Pediatric Diabetes 7: 274–278.

9. Smyth D, Cooper J, Howson J, Walker N, Plagnol V, et al. (2008) PTPN22 Trp620 explains the association of chromosome 1p13 with type 1 diabetes and shows a statistical interaction with HLA class II genotypes. Diabetes 57: 1730–1737.

10. Bjørnvold M, Undlien DE, Joner G, Dahl-Jørgensen K, Njøstad PR, et al. (2008) Joint effects of HLA, INS, PTPN22 and CTLA4 genes on the risk of type 1 diabetes. Diabetologia 51: 589–596.

11. Howson J, Barratt B, Todd J, Cordell H (2006) Comparison of population– and family–based methods for genetic association analysis in the presence of interacting loci. Genetic Epidemiology 29: 51–67.

12. Nejentsev S, Walker N, Riches D, Egholm M, Todd J (2009) Multiple rare variants in the virus receptor gene IFIH1 protect from autoimmune diabetes. Science In press.

13. Harjutsalo V, Podar T, Tuomilheto J (2005) Cumulative incidence of type 1 diabetes in 10,168 siblings of Finnish young–onset type 1 diabetic patients. Diabetes 54: 563–569.

14. Karvonen M, Tuomilheto J, Libman I, LaPorte R, the World Heath Oganization DIAMOND project group (1993) A review of recent epidemiological data on the worldwide incidence of type 1 (insulin-dependent) diabetes mellitus. Diabetologia 36: 883–892.

15. Hyttinen V, Kaprio J, Kinnunen L, Koskenvuo M, Tuomilheto J (2003) Genetic liability of type 1 diabetes and the onset age among 22,650 young Finnish twin pairs. Diabetes 52: 1052–1055.

16. Kyvik K, Green A, Beck-Nielsen H (1995) Concordance rates of insulin dependent diabetes mellitus: a population based study of young Danish twins. British Medical Journal 311: 913–917.

17. Pharoah PD, Antoniou A, Bobrow M, Zimmern RL, Easton DF, et al. (2002) Polygenic susceptibility to breast cancer and implications for prevention. Nature Genetics 31: 33–36.

18. Moore J, Gilbert J, Tsai CT, Chiang FT, Holden T, et al. (2006) A flexible computational framework for detecting, characterizing, and interpreting statistical patterns of epistasis in genetic studies of human disease susceptibility. Journal of Theoretical Biology 241: 252–261.

19. Anastasssious D (2007) Computational analysis of the synergy among multiple interacting genes. Molecular Systems Biology 3: 1–8.

20. Good I (1963) Maximum entropy for hypothesis formulation, especially for multidimensional contingency tables. The Annals of Mathematical Statistics 34: 911–934.

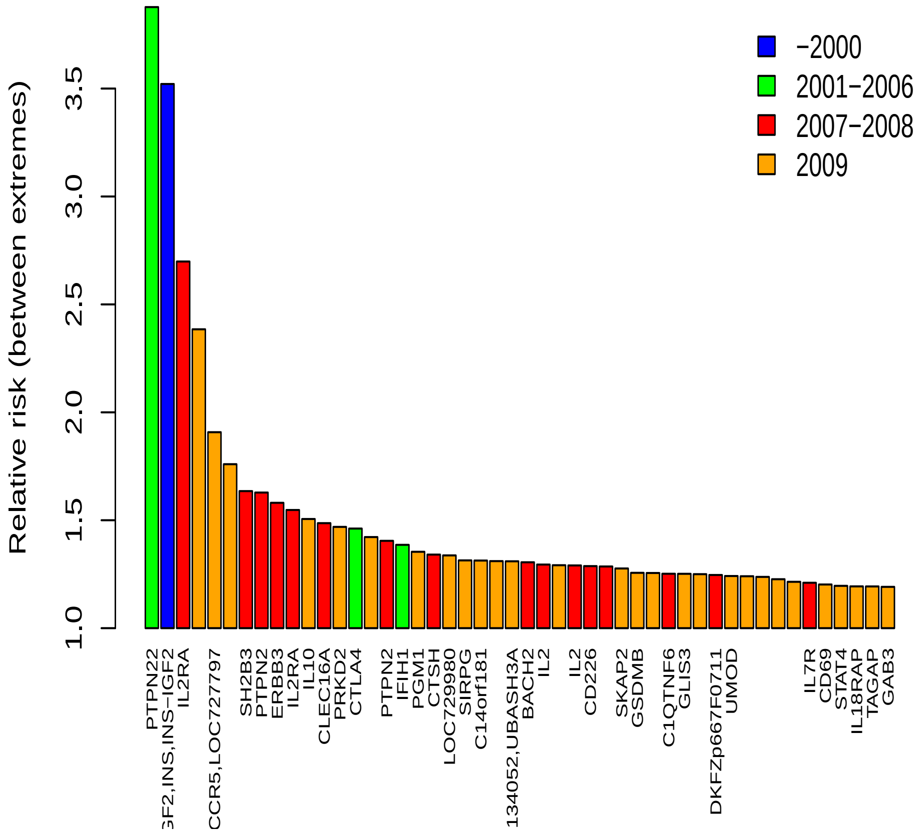

Supplementary Figure 1. Effect sizes for non-HLA SNP associations with T1D. The figure shows relative risk between the two homozygous genotypes. These estimates are based on data from up to 9,338 case and 11,303 controls, and use the model of multiplicative allelic effects when it fits the data and on the 2 df (genotype) model otherwise. Findings shown as “unpublished” are, at the time of writing, in press or submitted for publication. Gene names refer to the nearest gene within the region of LD surrounding the most associated SNP.

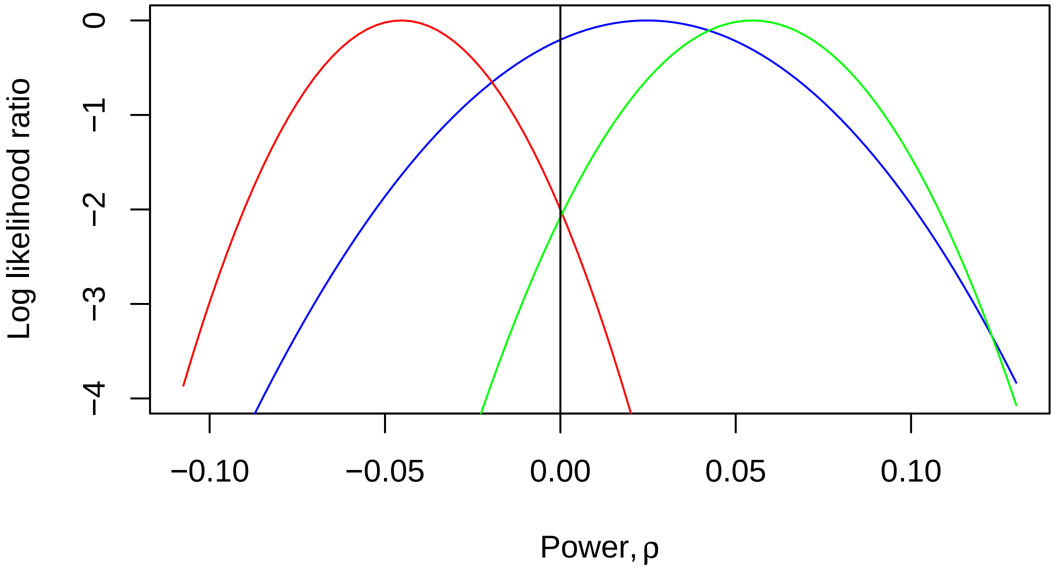

Supplementary Figure 2. Log likelihood profiles for the scale choice parameter, ρ. The blue curve refers to the model for the non-HLA SNPs in Supplementary Table 1 while the red curve is for the model for all SNPs. The green curve is for all SNPs but allows for dominance and interaction within the MHC region. Log likelihoods are expressed relative to the maximum likelihood estimate. The vertical line at $\rho = 0$ represents the multiplicative model; values to the right of this represent models in which effects accumulate less than multiplicatively while values to the left of the line represent accumulation more than multiplicatively.
